# Impact of population movement on the spread of 2019-nCoV in China

**DOI:** 10.1080/22221751.2020.1760143

**Published:** 2020-05-18

**Authors:** Chi Zhang, Cai Chen, Wei Shen, Feng Tang, Hao Lei, Yu Xie, Zicheng Cao, Kang Tang, Junbo Bai, Lehan Xiao, Yutian Xu, Yanxin Song, Jiwei Chen, Zhihui Guo, Yichen Guo, Xiao Wang, Modi Xu, Huachun Zou, Yuelong Shu, Xiangjun Du

**Affiliations:** a School of Public Health (Shenzhen), Sun Yat-sen University, Guangzhou, People’s Republic of China; b School of Public Health, Zhejiang University, Hangzhou, People’s Republic of China; c School of Intelligent Systems Engineering, Sun Yat-sen University, Guangzhou, People’s Republic of China; d Lingnan College, Sun Yat-sen University, Guangzhou, People’s Republic of China; e Key Laboratory of Tropical Disease Control (Sun Yat-sen University), Ministry of Education, Guangzhou, People's Republic of China

**Keywords:** 2019-nCov, population movement, spread, travel ban, risk

## Abstract

Since Dec 2019, China has experienced an outbreak caused by a novel coronavirus, 2019-nCoV. A travel ban was implemented for Wuhan, Hubei on Jan 23 to slow down the outbreak. We found a significant positive correlation between population influx from Wuhan and confirmed cases in other cities across China (R^2^ = 0.85, *P* < 0.001), especially cities in Hubei (R^2^ = 0.88, *P* < 0.001). Removing the travel restriction would have increased 118% (91%–172%) of the overall cases for the coming week, and a travel ban taken three days or a week earlier would have reduced 47% (26%–58%) and 83% (78%–89%) of the early cases. We would expect a 61% (48%–92%) increase of overall cumulative cases without any restrictions on returning residents, and 11% (8%–16%) increase if the travel ban stays in place for Hubei. Cities from Yangtze River Delta, Pearl River Delta, and Capital Economic Circle regions are at higher risk.

As of 14 February 2020, an outbreak caused by the novel coronavirus (2019-nCoV) has resulted in 66,492 confirmed cases across mainland China [1–3]. The majority of early domestic cases in other cities in China as well as international cases were associated with Wuhan [4–5]. Every year, billions of people travel across the country during the Chinese New Year (Spring Festival) holiday (celebrated from 10 January to 18 February for the year 2020) [6]. Wuhan is a transportation hub in central China with a population of 11 million [7] and an estimated five million people had already left the city as of 26 January 2020 [8]. The huge volume of population movement during the early stage of the epidemic, and the central role of transportation for Wuhan, facilitated the spread of the disease across China. Wuhan suspended all public transport in and out of the city on 23 January 2020 [9]. By 25 January, the first day of the Lunar New Year, thirty provinces, municipalities, and autonomous regions in mainland China have activated the highest level (Level-I) alert and response of public health emergency [10]. However, it is unclear to what degree Wuhan travel ban mitigated the epidemic size early on in other cities in China.

To quantitatively investigate the impact of population movement on the spread of 2019-nCoV, and estimate the effect of travel bans, we explored the relationship between population flux from Wuhan and cases in other cities one week later. We collected the number of laboratory confirmed cases from the daily official reports of the health commission of 34 provincial-level administrative units and 341 city-level units, from the time of the outbreak to 14 February 2020. And we used de-identified and aggregated real-time domestic population movement data (2019–2020) in China derived from Baidu Qianxi (http://qianxi.baidu.com). We assumed that confirmed cases in other cities between January 24 and 30 were imported from Wuhan based on the mean incubation period of one week [3], and thus correlated with the influx from Wuhan between January 17 and 23.

There is a statistically significant and strong correlation between the confirmed cases (from 24 January to 30 January 2020) and the population flow from Wuhan (from 17 January to 23 January 2020) for cities in China (R^2^ = 0.88, *P* < 0.001 for cities within Hubei province and R^2^ = 0.41, *P* < 0.001 for cities outside Hubei province, see Figure S1). We found the travel ban came after the outflow peak based on the trend in 2019 and dramatic movement reduction for Wuhan after the travel ban on 23 January 2020 (Figure 1(A)). We assumed that the observed case counts followed a Poisson distribution and built a simple linear regression model to simulate the effect of population flow on the imported cases (see Methods in supplementary materials for details). Based on this model, we then tested the consequences from different travel restriction policies (see Methods in supplementary materials for details). Overall, no restriction policy on travel potentially increased 118% (91%–172%) of the overall cases [13,857 (10,920–20,574) cases increased] for the coming week from 31 January to 6 February for all cities in China, compared with the implemented travel ban on 23 January (Figure 1(B) and Table S1). The top five cities in Hubei province, that is, Huanggang, Xiaogan, Jingzhou, Shennongjia, and Xianning, gained the most from the travel ban of Wuhan (Table S1). Had the travel ban come three days earlier, we would have seen an overall protection rate of 47% [(26%–58%), 3103 (1732–3830) cases reduced] for cases in the early stage of the outbreak (Figure 1(C)). Wenzhou, Chongqing, Shanghai, Beijing, Guangzhou, Shenzhen, and Hangzhou outside Hubei, and Huanggang, Xiangyang, Suizhou, Xiaogan, and Jingmen in Hubei province are among the top cities that would have benefited from an earlier lockdown (see Table S2 for details; also see Figure S2 and Table S3 for a week-earlier lockdown on Wuhan city).

**Figure 1.**
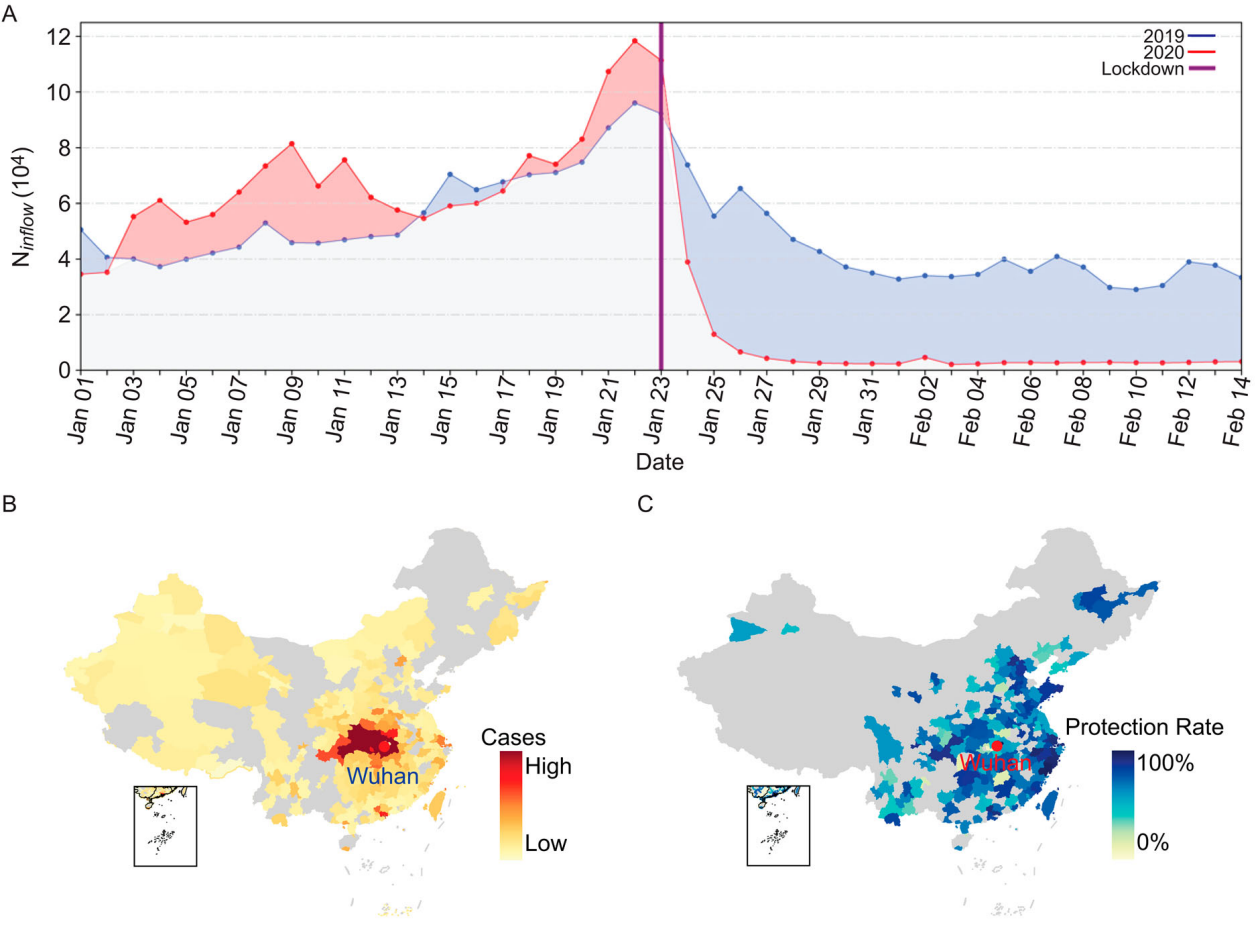
Impact of Wuhan’s travel ban on 2019-nCoV spread in China. (A) Population movement data for Wuhan between 1 January and 14 February 2020, and during the same lunar period of 2019. (B) Heat map of additional cases for cities in China, caused by cancelling Wuhan’s travel ban on 23 January 2020. (C) Protection rate heat map for cities in China based on a three-day-earlier lockdown of Wuhan. Protection rate was calculated as percentage of reduction from the observed cases. Cities without population influx from Wuhan (B) and without confirmed cases (C) between Jan 24 and Jan 30, 2020, are in grey (check supplementary materials for more details).

Based on the same assumption of the effect of population movement on the imported cases, cities with most imported risk are cities with the most population influx from high risk cities after the holiday (Figure S3A and Table S4). Overall, for the cities with positive population net reflux, we would expect a 61% (48%–92%) [30,490 (24,015–46,074) cases] increase of overall cumulative cases under the scenario without any restrictions on returning for work (Figure S3B and Table S5) and an 11% (8%–16%) [5275 (4188–7895) cases] increase of overall cumulative cases under the scenario with the travel ban for Hubei still in place (Figure S3C and Table S6).

Evaluating the impact of population movement on the spread of the new emerging 2019-nCoV virus is of crucial importance for targeted and precise public health response and control [11]. In summary, based on the simple model for imported cases, we studied the contribution of transportation and evaluated the consequences for different scenarios of travel bans and work resumption. Our findings suggest that population movement makes substantial contribution to the disease spread in the early stage of the outbreak and travel bans were effective but would have been more helpful if implemented earlier. Cities that are labor-intensive (e.g. Shanghai, Shenzhen, Guangzhou, and Beijing) need to take more strong measures to prevent potential rebound of 2019-nCoV outbreak incurred by population reflux. Beyond population movement restriction studied here, there are other factors that may contribute to the overall prevention and control of the epidemic, like the timely medical resources, socioeconomical factors and even climate factors etc., and should be systematically investigated in the future.
